# Naturally Acquired Transmission-Blocking Immunity Against Different Strains of *Plasmodium vivax* in a Malaria-Endemic Area in Thailand

**DOI:** 10.1093/infdis/jiad469

**Published:** 2023-11-07

**Authors:** Sataporn Thongpoon, Wanlapa Roobsoong, Wang Nguitragool, Sadudee Chotirat, Takafumi Tsuboi, Eizo Takashima, Liwang Cui, Tomoko Ishino, Mayumi Tachibana, Kazutoyo Miura, Jetsumon Sattabongkot

**Affiliations:** Mahidol Vivax Research Unit; Mahidol Vivax Research Unit; Department of Molecular Tropical Medicine and Genetics, Faculty of Tropical Medicine, Mahidol University, Bangkok, Thailand; Mahidol Vivax Research Unit; Division of Malaria Research, Proteo-Science Center, Ehime University, Matsuyama, Japan; Division of Malaria Research, Proteo-Science Center, Ehime University, Matsuyama, Japan; Department of Internal Medicine, Morsani College of Medicine, University of South Florida, Tampa, FL, USA; Department of Parasitology and Tropical Medicine, Tokyo Medical and Dental University, Tokyo, Japan; Division of Molecular Parasitology, Proteo-Science Center, Ehime University, Toon, Ehime, Japan; Laboratory of Malaria and Vector Research, National Institute of Allergy and Infectious Diseases, National Institutes of Health, Rockville, Maryland, USA; Mahidol Vivax Research Unit

**Keywords:** heterogeneity, malaria, naturally acquired immunity, *Plasmodium vivax*, transmission-blocking immunity (TBI)

## Abstract

**Background:**

Human immunity triggered by natural malaria infections impedes parasite transmission from humans to mosquitoes, leading to interest in transmission-blocking vaccines. However, immunity characteristics, especially strain specificity, remain largely unexplored. We investigated naturally acquired transmission-blocking immunity (TBI) against *Plasmodium vivax*, a major malaria parasite.

**Methods:**

Using the direct membrane-feeding assay, we assessed TBI in plasma samples and examined the role of antibodies by removing immunoglobulins through protein G/L adsorption before mosquito feeding. Strain specificity was evaluated by conducting a direct membrane-feeding assay with plasma exchange.

**Results:**

Blood samples from 47 patients with *P vivax* were evaluated, with 37 plasma samples successfully infecting mosquitoes. Among these, 26 showed inhibition before immunoglobulin depletion. Despite substantial immunoglobulin removal, 4 samples still exhibited notable inhibition, while 22 had reduced blocking activity. Testing against heterologous strains revealed some plasma samples with broad TBI and others with strain-specific TBI.

**Conclusions:**

Our findings indicate that naturally acquired TBI is mainly mediated by antibodies, with possible contributions from other serum factors. The transmission-blocking activity of plasma samples varied by the tested parasite strain, suggesting single polymorphic or multiple targets for naturally acquired TBI. These observations improve understanding of immunity against *P vivax* and hold implications for transmission-blocking vaccine development.

## Teaser

Natural malaria infections trigger human immunity against parasite transmission to mosquitoes. We studied transmission-blocking immunity in 47 patients with *Plasmodium vivax*. Antibodies primarily mediated transmission-blocking immunity, with varying effectiveness against different parasite strains, offering insights for vaccine development.

Malaria is a public health problem in tropical and subtropical regions. Although a significant reduction in the disease burden has been observed in many places, challenges exist in others, leading to the stalling of the progress toward elimination [1]. In sub-Saharan Africa, *Plasmodium falciparum* is the predominant malaria parasite species. However, *Plasmodium vivax* is more common in many other places, including Southeast Asia, Meso- and South America, and Oceania [2]. *P vivax* is generally less virulent than *P falciparum* [3], yet *P vivax* possesses a few biological features that allow it to withstand malaria interventions better. First, *P vivax* forms hypnozoites, a latent form of the parasite in the liver, which can persist for months to years in the human body after the initial inoculation by mosquito bites. Second, the development of gametocytes of *P vivax* is much faster than that of *P falciparum*. Third, *P vivax* can develop inside the mosquito vectors at a lower temperature and more quickly than *P falciparum* [4]. Thus, to accelerate vivax malaria elimination, focused attacks with novel interventions, including measures to interrupt *P vivax* transmission, are deemed essential [5, 6].

In malaria-endemic areas, natural malaria infections of *P falciparum* and *P vivax* can induce transmission-blocking immunity (TBI) [7–10], which reduces the transmission of the parasites from humans to *Anopheles* mosquitoes [11]. Because strong naturally acquired transmission-blocking activities have been observed [12], invoking or enhancing TBI via vaccination is one approach recognized by many to accelerate malaria elimination [13]. Vaccine-induced TBI is considered to be mediated by antibodies [14], but the targets of naturally acquired TBI have not been fully unveiled. In the case of *P falciparum*, multiple immune-epidemiology studies have shown significant correlations between anti-Pfs48/45 or anti-Pfs230 antibody titers and TBI [8]. However, Stone et al reported that Pfs48/45- and Pfs230-depleted antibodies from malaria-endemic areas still maintained TBI and antibody titers against 43 novel proteins were also associated with TBI [15]. The results suggest that there could be unidentified transmission-blocking vaccine (TBV) candidates. In the case of *P vivax*, a recent study revealed that antibodies against Pvs47, Pvs230, Pvs25, and PvHAP2 were associated with naturally acquired TBI [16]. In addition to these antigens, Pvs28 has been considered a promising TBV candidate [17], although the naturally acquired TBI against Pvs28 has never been investigated. Considering a TBV, strain transcendency is one of the key factors that can have a significant impact on vaccine efficacy in the field. Yet, strain specificity or transcendency of naturally acquired TBI is poorly understood, especially for *P vivax*.

In this study, TBI in the immune plasma of patients with *P vivax* in Thailand was evaluated by direct membrane-feeding assay (DMFA). To determine whether TBI in the plasma samples was mediated by immunoglobulin (Ig), DMFA was conducted with whole plasma and Ig-depleted plasma samples. In addition, the plasma samples were tested with heterologous *P vivax* gametocytes collected from different patients to evaluate the strain specificity. Last, to evaluate the role of specific Ig targets in natural immunity, the association between TBI and antibody titers against 3 TBV candidates (Pvs25, Pvs28, and Pvs230) was examined.

## MATERIALS

### Parasite Isolate Collection

The study was conducted from May 2016 to December 2018, involving malaria cases presenting at health care facilities (hospital and malaria clinics) in Thasongyang district, Tak (northwestern of Thailand), and Bannang Sata district, Yala (southern of Thailand). Species identification was done by a microscopist, and patients infected with *P vivax* alone were enrolled. The blood from patients with malaria was collected into a heparinized tube and transferred to the field laboratory, with the temperature kept at ∼37 °C. Written informed consent was received from all volunteers before enrollment. This study was performed under protocols approved by the Ethical Review Committee of the Faculty of Tropical Medicine, Mahidol University, Thailand (MUTM 2011-040-05, MUTM 2011-040-06, MUTM 2016-031-02).

### Direct Membrane-Feeding Assay

Malaria-infected blood was divided into 3 tubes (500 µL/1.5-mL tube). Three DMFAs were conducted with autologous parasites: whole blood, AB serum replacement, and Ig-depleted plasma. For the whole blood DMFA, whole blood without any treatment was fed to starved 5- to 7-day-old laboratory-reared *Anopheles dirus* mosquitoes (100/cup) for half an hour, and unfed mosquitoes were subsequently removed.

For the AB serum replacement DMFA, the patient's blood was centrifuged to separate the plasma from the packed cells. The plasma was removed, and the packed cells were washed with RPMI1640 medium. The packed cells were reconstituted with the AB serum of healthy volunteers from the Thai Red Cross Society and then fed to *A dirus* mosquitoes as indicated. For the Ig-depleted DMFAs, the patient's plasma was isolated as previously detailed and then passed through Pierce protein L and G agarose resin (Thermo Fisher Scientific) to deplete the Ig, following manufacturer's instructions. The Ig-depleted plasma and washed packed cells were mixed and fed to the mosquitoes. The farthest clinic was ∼165 km away from the field laboratory, and it took around 4 to 6 hours from blood collection to DMFA. The process time for IgG/IgM depletion was ∼45 minutes, and the blood samples were kept at ∼37 °C during the entire process except centrifugation, which was done at room temperature. For the heterologous DMFA, isolated patients’ plasma samples or healthy AB serum (stored at −80 °C and thawed immediately before use) were mixed with washed packed cells from different samples of *P vivax* blood and then fed to the mosquitoes.

For all DMFAs, mosquitoes were dissected (n = 20 per group) 7 to 9 days after the feeds to determine the oocyst density (oocyst number/midgut in all midguts dissected).

### Enzyme-Linked Immunosorbent Assay

To detect the efficiency of IgG or IgM depletion in the protein L/G–treated plasma samples, 100 µL of 1:10 000 diluted plasma samples (before and after treatment) were coated on a 96-well plate and incubated overnight at 4 °C. After 3 washes with 200 μL of PBST (1× PBS, 0.1% Tween 20) each, the wells were blocked with 200 μL of 10% skim milk in PBST for 1 hour. Then, 100 µL of 1:2500 diluted *horseradish peroxidase*–conjugated anti-human IgG or IgM (Merck Ltd) was added and incubated for 1 hour. After 3 washes with 200 μL of PBST each and 3 rinses with 200 μL of PBS each, 100 µL of ABTS peroxidase substrate (2,2′-azinobis[3-ethylbenzothiazoline-6-sulfonic acid]; Merck Ltd) was added. The optical densities at 405 nm (OD_405_) were determined for 45 minutes on a spectrophotometer (Synergy Hybrid Reader). The percentage reduction of IgG or IgM in each plasma sample was calculated as 100 – [(OD_405_ after treatment/OD_405_ before treatment) × 100].

### Measurement of the Immunoreactivity by Luminex

As well-known TBV candidates of *P vivax*, recombinant Pvs25 and Pvs28 and a fragment of Pvs230 proteins were expressed via a previously described wheat germ cell–free expression system (CellFree Sciences) [18]. Briefly, a DNA fragment corresponding to amino acid region A_23_–L_195_ of Pvs25 (PVX_111175) with a C-terminal hexahistidine tag was cloned into wheat germ cell–free expression vector pEU (CellFree Sciences). Similarly, Pvs28 (PVX_111180: amino acid K_23_–S_214_) and a region of Pvs230 (PVX_003905: amino acid V_236_–T_943_) were also cloned into the pEU vector. These 3 recombinant proteins were expressed with the wheat germ cell–free expression system and Ni affinity purified. Purified recombinant proteins were coupled to Bio-Rad COOH microspheres (0.5 μg) [19]. Luminex assays were performed to measure IgG antibodies from patients with malaria against these sexual-stage antigens [19]. Briefly, 50 μL of 1:100 diluted patient's plasma was incubated with 50 μL of protein-conjugated microspheres (500 coated beads) in a 96-well plate on a plate shaker at ambient temperature. A pool of AB serum of healthy volunteers (∼100) from the Thai Red Cross Society was tested at 1:100 dilution as a negative control. Plasma specimens obtained from hyperimmune individuals in Papua New Guinea were pooled and used as the positive control from which 2-fold serially diluted standard curves were established, as previously described [20]. The microspheres were washed 3 times with 100 μL of PBST and incubated with 1:100 dilution of phycoerythrin-conjugated anti-human IgG (Jackson Immuno Research Laboratories) for 15 minutes on a plate shaker at ambient temperature. After washing, the conjugated microspheres were resuspended in 100 μL of PBST. The assay was run on a Bio-Plex 200 (Bio-Rad Laboratories Ltd), and median fluorescent intensity (MFI) was obtained.

### Statistical Analysis

When a mean oocyst density in the AB serum feed was <2.5, the blood (either autologous or heterologous DMFA) was considered noninfectious and thus removed from the analysis.

The TBI of the whole blood due to antibodies and other factors against autologous parasites (ie, from the same patients) or heterologous parasites (ie, from different patients) was calculated as follows:

percentage transmission-reducing activity (%TRA) = [1 – (mean oocyst density_whole blood_/mean oocyst density_AB serum_)] × 100.

Similarly, the TBI of the Ig-depleted plasma (tested with autologous parasites only) was calculated as follows:

%TRA = [1 – (mean oocyst density_Ig-depleted plasma_/mean oocyst density_AB serum_)] × 100.

The 95% CI and *P* value of each %TRA was calculated by a zero-inflated negative binomial model as described previously [21]. In addition to the %TRA values, the log of the mean oocyst ratio (LMR) was calculated as follows:


$$\begin{array}{r} \mathrm{LMR}=\mathrm{lo}g_{10}(\;\mathrm{mean}\;\mathrm{oocyst}\;\mathrm{densit}y_{\mathrm{control}}/\;\mathrm{mean}\;\mathrm{oocyst}\;\mathrm{densit}y_{\mathrm{test}} \end{array}\;).$$

LMR and %TRA are different ways of transformation, and LMR = log_10_ [100/(100 – %TRA)]. When LMR > 2 (TRA > 99%), the value is assigned as an LMR of 2 for the analysis. Based on the zero-inflated negative binomial model, a TRA of approximately 60% (or −150%), equivalent to an LMR of approximately ∼0.4 (or −0.4), is considered the threshold level to reach statistical significance (significant inhibition or enhancement) when *P vivax* DMFA is conducted in the assay condition described in this study.

A Wilcoxon matched-pairs signed rank test was used to compare inhibition levels before and after Ig depletion, and a Spearman rank test was used to determine correlations between data sets.

Statistical data were analyzed with Prism (version 9.0; GraphPad) or R (version 4.2.1; R Foundation for Statistical Computing), and differences were considered significant when *P* < .05.

## RESULTS

### TRA Profiles of Blood Samples From *P vivax* Cases Against Autologous Parasites

We collected 47 blood samples from patients with acute *P vivax* (18–62 years old, 39 males and 8 females) and used them in the DMFA. Of the 47 cases, 5 did not infect mosquitoes at all under whole blood and AB serum replacement conditions, and another 5 had an average <2.5 oocysts per mosquito under both conditions. We excluded the 10 cases because an accurate inhibition or enhancement measurement has shown to be difficult in an assay with lower oocysts in the negative control group [21]. For the remaining 37 cases that were analyzed in this study, the median (IQR) parasite density per microliter was 142 (1–357) rings, 316 (126–745) trophozoites, 1 (0–20) schizonts, 19 (8.5–36) male gametocytes, and 33 (14.5–70) female gametocytes. The total gametocyte count was 56 (24–117.5) and the total parasite count was 733 (406–1284). All patients had uncomplicated malaria. The proportion of febrile cases, defined as individuals with a body temperature ≥37.5 °C at the time of blood draw, was 41% (15/37), while that of nonfebrile cases was 59% (22/37). The parasite density, stage distribution, and patient body temperature and age are available in Supplementary Table 1.

**Figure 1.**
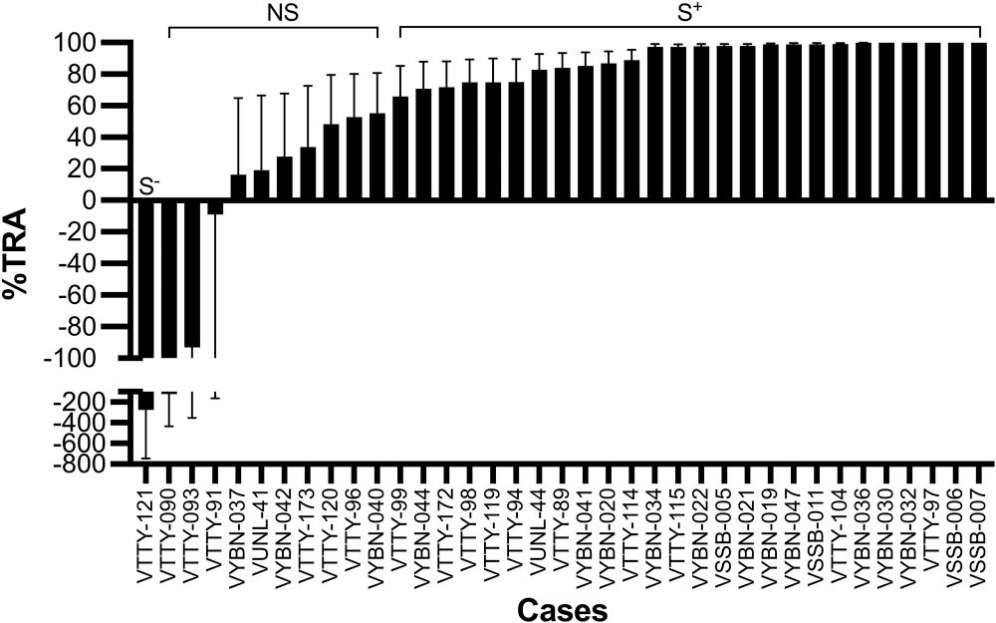
The transmission-reducing activity of *Plasmodium vivax* infection against autologous parasites. Direct membrane-feeding assay was conducted with plasma samples from 37 patients infected with *P vivax* against autologous parasites. Data are presented as the percentage of transmission-reducing activity (TRA) of a *P vivax* infection with 95% CI. Participants were categorized into 3 groups: NS, insignificant inhibition; S^+^, significant inhibition; S^−^, significant enhancement.

Of the 37 cases analyzed, the median (IQR) oocyst density in the AB-negative control groups was 70.0 (14.2–168.7) with an infection prevalence of 94.9% (91.4%–96.8%), and corresponding values in the whole blood DMFA were 11.4 (1.9–42.8) and 75% (1%–95%; including autologous and heterologous assays). The average oocyst density in each assay for each test condition is presented in Supplementary Table 1, and the frequency distribution of oocyst densities is displayed in Supplementary Figure 1. The plasma samples displayed various %TRA, ranging from complete inhibition to substantial enhancement of mosquito infectivity. Of the 37 cases, 26 exhibited statistically significant inhibitions, and 5 completely blocked mosquito infections (100% TRA). Of the 37 cases, 10 had insignificant inhibitions. However, only 1 case had significant transmission enhancements (Figure 1 and Supplementary Figure 2). We compared %TRA among the 4 blood groups (A, B, O, or AB) but did not find significant differences (Supplementary Figure 3).

**Figure 2.**
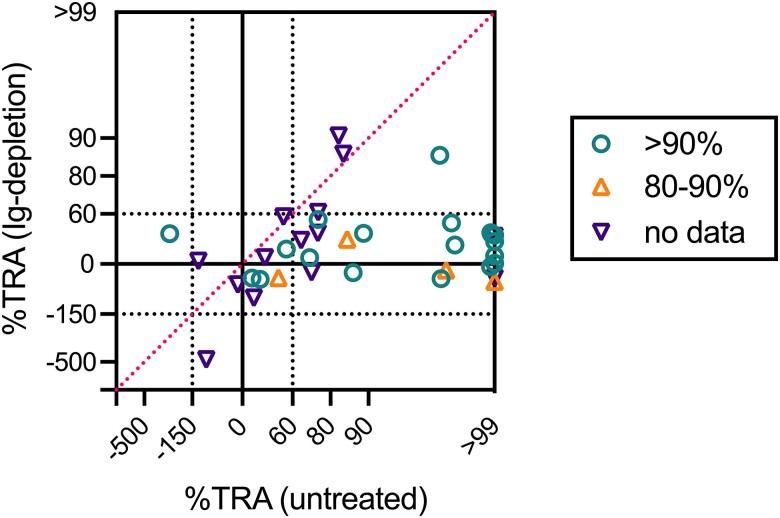
The transmission-reducing activity (TRA) against autologous parasites before and after immunoglobulin (Ig) depletion. An additional direct membrane-feeding assay was conducted with protein L/G–treated plasma samples (Ig-depleted plasma). Each sample shows the percentage TRA of whole blood (untreated plasma, x-axis) and Ig-depleted plasma (y-axis). Data are color coded by level of reduction (percentage) in IgG signal after protein L/G treatment for 23 cases; percentage reduction values were not measured for 14 cases due to the available volume of samples. While the figure is drawn per an LMR scale (log of mean oocyst ratio), corresponding percentage TRA levels are shown for easiness. Any LMR values >0.40 (TRA >60%) are considered statistically significant inhibitions (vs no inhibition), and LMR values < −0.40 (TRA < −150%) are statistically significant enhancements (black dotted lines) based on the zero-inflated negative binomial model (ZINB model) [21]. The red dotted line denotes *y* = *x*.

To assess whether the TBI seen in the whole blood as compared with the control AB serum was due to Igs in the patients’ plasma samples, an additional DMFA was conducted with protein L and G–treated plasma samples (Ig-depleted plasma samples). The IgG and IgM concentrations before and after Ig depletion treatment for 23 paired plasma samples were determined (ie, for which enough volume was available), and the Ig depletion led to a median reduction of 97% (IQR, 80%–99%) in IgG and 72% (41%– 91%) in IgM (Supplementary Figure 4). The percentage reduction results for individual samples are presented in Supplementary Table 1. The data indicated that most IgG and more than half of IgM fractions were removed from the original (untreated) plasma samples. The median (IQR) oocyst density of the Ig-depleted group was 53.4 (18.1–137), and the frequency distribution of oocyst density is displayed in Supplementary Figure 1. When the %TRA of untreated and Ig-depleted plasma samples was compared (Figure 2), Ig-depleted plasma samples had significantly lower inhibitions overall, as predicted (*P* < .0001, Wilcoxon matched-pairs signed rank test). Of the 26 samples that showed significant inhibitions with the untreated plasma samples, 22 demonstrated insignificant inhibitions after the treatment. Interestingly, the other 4 samples demonstrated similar and significant inhibitions even after the Ig depletion; the TRA before and after treatment was 97% to 86%, 84% to 87%, 83% to 90%, and 75% to 61%. The results strongly suggest that a proportion of plasma samples have IgG-independent TBI and possibly IgM-independent TBI.

### TRA of Plasma Samples in Heterologous Feeding

**Figure 3.**
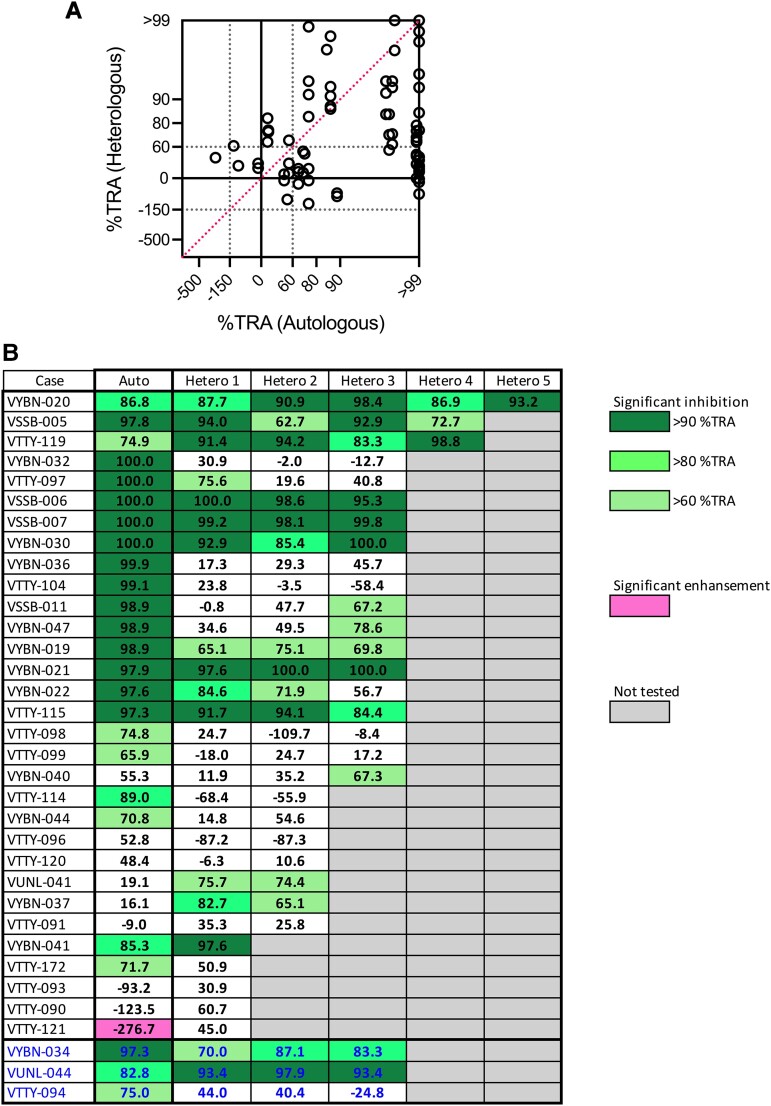
The transmission-reducing activity (TRA) against heterologous parasites. An additional direct membrane-feeding assay was conducted via heterologous parasites with untreated plasma samples (n = 34). The percentage TRA levels for autologous and heterologous parasites (1–5 heterologous parasites) are shown in the same way as Figure 2 (*A*) or in tabular form (*B*). *A*, Data from the individual heterologous parasites are shown as different dots. For example, there are 5 VYBN-020 data points with the same 86.8% TRA autologous value in the figure. Plasma samples that showed immunoglobulin G–independent TBI (VYBN-034, VUNL-044, and VTTY-094) are omitted in panel *A*, but they are included in blue letters in panel *B*.

For 34 plasma samples, we performed heterologous feeding experiments to determine whether TBI varied among *P vivax* clinical isolates (Figure 3). For the heterologous feeds, only untreated plasma samples were utilized, due to the limitation of volume available. The number of heterologous DMFA experiments ranged from 1 to 5 and an average of 2.6 per plasma. When IgG-independent TBI samples were excluded, there was a weak but significant correlation between %TRA in autologous and heterologous parasites overall (Spearman rank coefficient, 0.223; *P* = .046; Figure 3*A*). However, strong heterogeneity was observed in individual levels (Figure 3*B*). This analysis showed that while some patient samples had broad TBI against a wide range of parasite isolates, some possessed only strain-specific TBI, and others had insignificant TBI for any of the isolates tested. VTTY-121, which showed significant enhancement for autologous parasites (−276.7% TRA, *P* < .001), was tested against 1 heterologous isolate and demonstrated insignificant inhibition (45.0% TRA, *P* = .237). The plasma samples with IgG-independent TBI also had different inhibition patterns against heterologous parasites (blue letters in Figure 3*B*; VTTY-089 was not tested for the heterologous DMFA).

### Correlation of TRA With IgG Responses Against *P vivax* Sexual-Stage TBV Candidates

**Figure 4.**
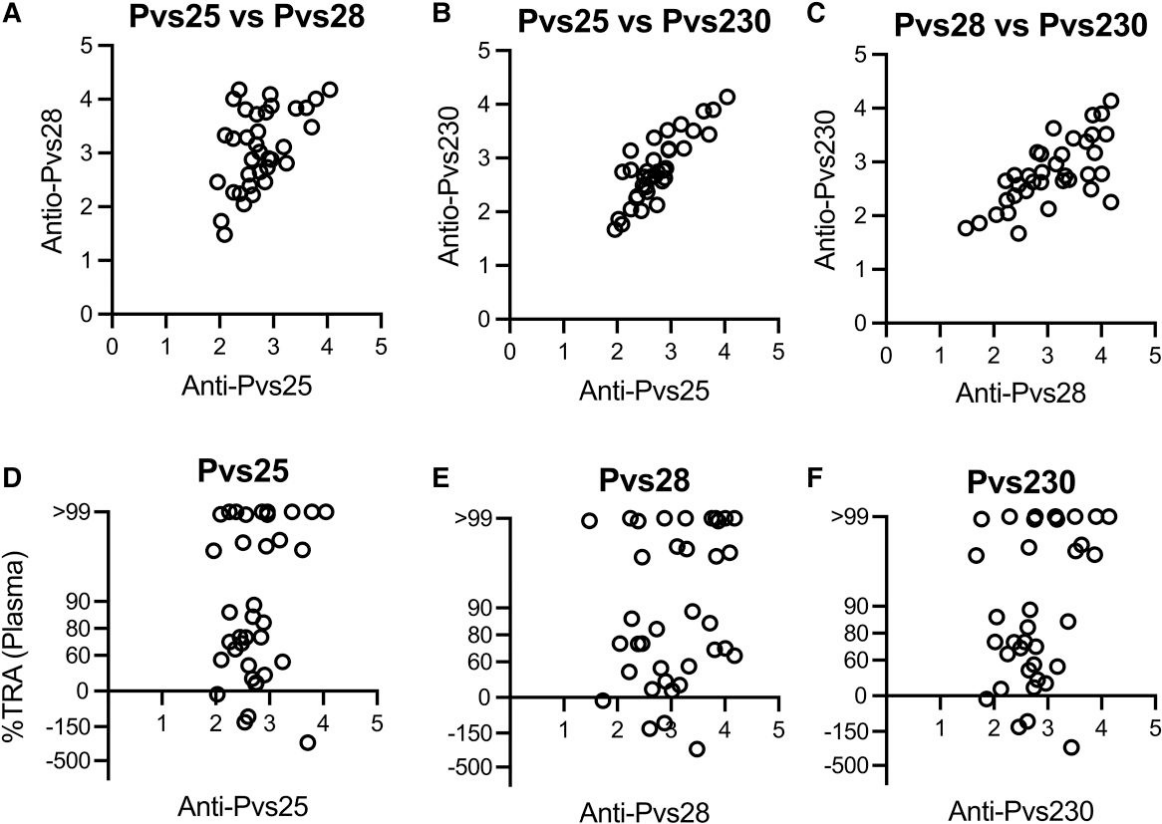
Immunoreactivity of antibodies against the Pvs25, Pvs28, and Pvs230 proteins. The antibody level in the plasma of each patient with malaria was measured against *Plasmodium vivax* sexual-stage proteins: Pvs25, Pvs28, and Pvs230. Log-transformed mean fluorescence intensity (MFI) values of antibody responses are presented. A negative control (a pool of AB sera from healthy Thai volunteers) resulted in 0.84, 0.57, and 0.73 log-MFI values for Pvs25, Pvs28, and Pvs230, respectively. There are significant correlations in the MFI values among antigens: *A*, *P* = .0046; *B* and *C*, *P* < .0001 (Spearman rank test). The correlations between the MFI values against the individual antigens and the percentage transmission-reducing activity (TRA) of autologous parasites are shown: *D*, *P* = .288; *E*, *P* = .181; *F*, *P* = .051 (Spearman rank test).

We used the Luminex assay to determine the IgG responses of the patients’ plasma with 3 sexual-stage antigens: Pvs25, Pvs28, and Pvs230. The log-transformed MFI values were used as a proxy of antigen-specific antibody titers. There were significant correlations among the MFI values of the 3 antigens (Figure 4*A*-*C*; *P* < .005 for all, Spearman rank test). Next, the correlation between the values and corresponding %TRAs against autologous parasites of individual patients’ plasma samples was examined for each antigen (Figure 4*D*-*F*). There were insignificant correlations for all of them by a Spearman rank test, whereas the *P* value for Pvs230 was approaching significance (*P* = .051). Finally, the association between strain specificity or transcendency in DMFA and antibody titers was examined (Supplementary Figure 5). There was no obvious relationship between DMFA and Luminex results. For example, VYBN-020, with >80% TRA in DMFA against autologous and all 5 heterologous parasites tested (ie, strain-transcending TBI), had lower titers against all 3 antigens. Yet, VTTY-104, with >80% TRA against autologous but <24% TRA against all 3 heterologous parasites tested (ie, strain-specific TBI), had higher titers against all 3 antigens.

## DISCUSSION

Naturally acquired TBI against *P falciparum* has been studied extensively [9, 15, 22, 23] but not against *P vivax*. This study revealed the following: (1) while most TBI in *P vivax* blood samples from Thai patients was attributed to Ig, ∼15% of the samples showed at least IgG-independent TBI; (2) there was considerable heterogeneity of TBI against different *P vivax* gametocytes.

IgG in the blood is considered responsible for the inhibitory activity; thus, researchers have examined correlations between IgG titers against malaria antigens and DMFA results [24–27]. However, the validity of the assumption had not been evaluated experimentally before. While the majority of samples (∼85%) had lower %TRA levels after the protein L/G treatment (where 97% and 72% of IgG and IgM were removed, respectively), ∼15% of blood samples still demonstrated significant and very similar %TRA levels before and after the treatment. To our knowledge, this is the first study that compared paired whole and Ig-depleted plasma samples in a DMFA. Such IgG-independent TBI could be explained by other blood factors, such as cytokines and complement, which have been shown to block gametocyte transmission to mosquitoes [12–14, 28] and/or non-IgG Igs (eg, remaining IgM, IgA, IgE). While the responsible factors for the IgG-independent TBI were not investigated in this study, if a researcher wants to determine IgG-dependent TBI accurately, DMFA should be conducted with purified IgG, instead of whole plasma/serum.

A large heterogeneity of TBI against different *P vivax* gametocytes was seen in this study. It seems that neither the breadth nor the intensity of antibody responses against individual Pvs25, Pvs28, and Pvs230 antigens could explain the TBI. While the antibody titers against 3 antigens strongly correlated—as noted in a recent study by Tebeje et al [16], where strong correlations were seen among different antigens (including *P vivax* blood- and sexual-stage antigens)—some high-blocking, strain-transcending, and IgG-dependent plasma samples (eg, VYBN-020 and VTTY-119) had relatively lower MFI values vs all 3 antigens. It is challenging to determine the mechanisms of naturally acquired TBI. However, further investigation for the IgG-dependent and strain-transcending plasma samples, especially with lower titers against known TBV candidates, could benefit novel TBV candidate discovery.

Transmission enhancement activity in naturally acquired immunity has also been studied [29–31]. We found statistically significant enhancement in 1 of 37 cases [−277% TRA]. The result must be interpreted carefully. First, the formula used to calculate %TRA gives a different impression. For example, if average oocysts in groups A and B are 10 and 5, respectively, the %TRA of group B against group A is 50 = [1 – [5/10]] × 100, while the %TRA of group A against group B is −100 = [1 – [10/5]] × 100. Although −100 looks to have a greater effect than 50, both mean that there was a 2-fold difference in oocyst density between the groups. If a researcher thinks that only >80% TRA is a “true” inhibition (5-fold difference between test and control groups), then only < −400% TRA is a “true” enhancement. Second, 37 autologous parasite comparisons should be interpreted with the family-wise error rate. Suppose that *P* < .05 is used as a significant threshold (*P* < .025 for significant inhibition and *P* < .025 for significant enhancement), there is a 61% chance that at least 1 of 37 samples would show “significant enhancement.”

There are several limitations in this study. There was a limitation of blood samples that could be collected from patients per time, so each assay condition was tested once. Variability in DMFA is known to be large, especially in samples that have weak activities [21]. Therefore, the binomial assessment was utilized, as statistically significant (ie, the samples are likely to have true activities) or insignificant (ie, the samples may or may not have weak activities). Second, we did not collect DNA samples from the parasites. Therefore, we could not assess whether or how much the heterogeneity in DMFA results against different isolates can be explained by sequence polymorphism in potential target antigens. Similarly, it is not clear whether recombinant proteins used for the Luminex assay did or did not have the same sequences in the parasites. Third, since infectivity in AB serum–replaced feeds (negative controls) varied significantly among other patients’ blood (average, 3–515), only TRA (inhibition in oocyst density) was used for the analysis, as transmission-blocking activity (inhibition in prevalence of infected mosquitoes) changes dramatically depending on the infectivity in the control even when the same sample is tested [21].

Naturally acquired TBI against *P vivax* has been evaluated in only a few studies. This study aimed to identify and characterize immunity to *P vivax* in Thailand. The 2 novel findings from this study should be considered when one interprets results from *P vivax* DMFA studies and/or designs future studies.

## Supplementary Data

Supplementary materials are available at *The Journal of Infectious Diseases* online (http://jid.oxfordjournals.org/). Supplementary materials consist of data provided by the author that are published to benefit the reader. The posted materials are not copyedited. The contents of all supplementary data are the sole responsibility of the authors. Questions or messages regarding errors should be addressed to the author.

## Supplementary Material

jiad469_Supplementary_Data

## Notes

***Acknowledgments***. We thank all of the patients who participated in this study, as well as the field staff, insectary staff, and administrative staff of the Mahidol Vivax Research Unit, Faculty of Tropical Medicine, Mahidol University.

***Author contributions***. S. T.: conducting experiments, collecting data, analyzing the results, and writing the manuscript. W. R.: providing technical or material support for the study, analysis and interpretation of data. W. N.: analysis and interpretation of data, revising the manuscript. S. C.: providing technical or material support for the study. T. T.: conception and design of the study. E. T.: conception and design of the study. L. C.: providing funding or resources for the study. T. I.: conception and design of the study. M. T.: conception and design of the study. K. M.: analysis and interpretation of data, drafting or revising the manuscript. J. S.: conception and design of the study, providing technical or material support for the study, and approval of the final version of the manuscript for publication.

***Disclaimer***. The funders had no role in study design, data collection and analysis, the decision to publish, or manuscript preparation.

***Financial support***. This work was supported by the National Institute for Allergy and Infectious Diseases and the Fogarty International Center at the National Institutes of Health (grants U19 AI089672 and D43TW006571); Fundamental Fund 2023- FF66; the intramural program of the National Institute of Allergy and Infectious Diseases (to K. M.); and the JSPS-KAKENNHI (19KK0206 to T. I.).
