# Supplementary material for: Naturally Acquired Transmission-Blocking Immunity Against Different Strains of Plasmodium vivax in a Malaria-Endemic Area in Thailand
Source: J Infect Dis. 2023 Nov 7;229(2):567–75. doi: 10.1093/infdis/jiad469 (PMC10873188; doi:10.1093/infdis/jiad469)
Supplement: jiad469_Supplementary_Data [file jiad469_supplementary_data.zip › P_vivax_transmission_blocking_immunity_28Sep23.docx]

**Supplementary Figures legends**

**Supplementary Figure S1. Frequency Distribution in Direct Membrane Feeding Assay (DMFA) under Different Conditions.** The graph illustrates the results of DMFA performed on thirty-seven *P. vivax*-infected blood samples, divided into three subgraphs (A, B, C). Each subgraph represents a specific condition: AB-DMFA (AB serum-replacement), WB-DMFA (whole blood), and IG-DMFA (Immunoglobulin depletion). The frequencies shown represent the log oocyst number per mosquito, indicating successful infections by the blood samples under each condition.

**Supplementary Figure S2. Mosquito infectivity of the thirty-seven *P. vivax* specimens with and without AB serum replacement.** Thirty-seven *P. vivax*- infected blood samples were tested in direct membrane feeding assays under the whole blood (WB-DMFA) and AB serum-replacement (AB-DMFA) conditions. Paired log mean oocyst/gut (A) and prevalence of infection (B) are shown.

**Supplementary Figure S3. Transmission-reducing activity classified by blood groups.** Percent TRA of 37 individuals grouped by blood groups. A long horizontal bar designates the median number of %TRA in each group, and two short horizontal bars represent the interquartile range in each group. There was an insignificant difference among 4 blood groups (*p*=0.737 by a Kruskal-Wallis test).

**Supplementary Figure S4. Reduction of antibodies level in the immunoglobulin-depleted plasma.** The antibody level of patients’ plasma was compared before and after Ig-depletion. Percent reductions of two main isotypes of individuals were represented; Red dots designate IgG, and blue dots designate IgM. The long and short horizontal bars designate the median and the interquartile range in each group, respectively. There was a significant difference in % Ig-reduction between IgG and IgM (*p*<0.0001 by a Wilcoxon matched-pairs signed rank test).

**Supplementary Figure S5. Correlation between strain-specificity in DMFA and antibody titers.** Left side of panel shows DMFA results, which are the same as Figure 3B, and right side shows Luminex results. For each test antigen, cases are color coded by the quartile range in a total of 36 cases tested by Luminex.
