# Supplementary figures and images for: Naturally Acquired Transmission-Blocking Immunity Against Different Strains of Plasmodium vivax in a Malaria-Endemic Area in Thailand
Source: J Infect Dis. 2023 Nov 7;229(2):567–75. doi: 10.1093/infdis/jiad469 (PMC10873188; doi:10.1093/infdis/jiad469)

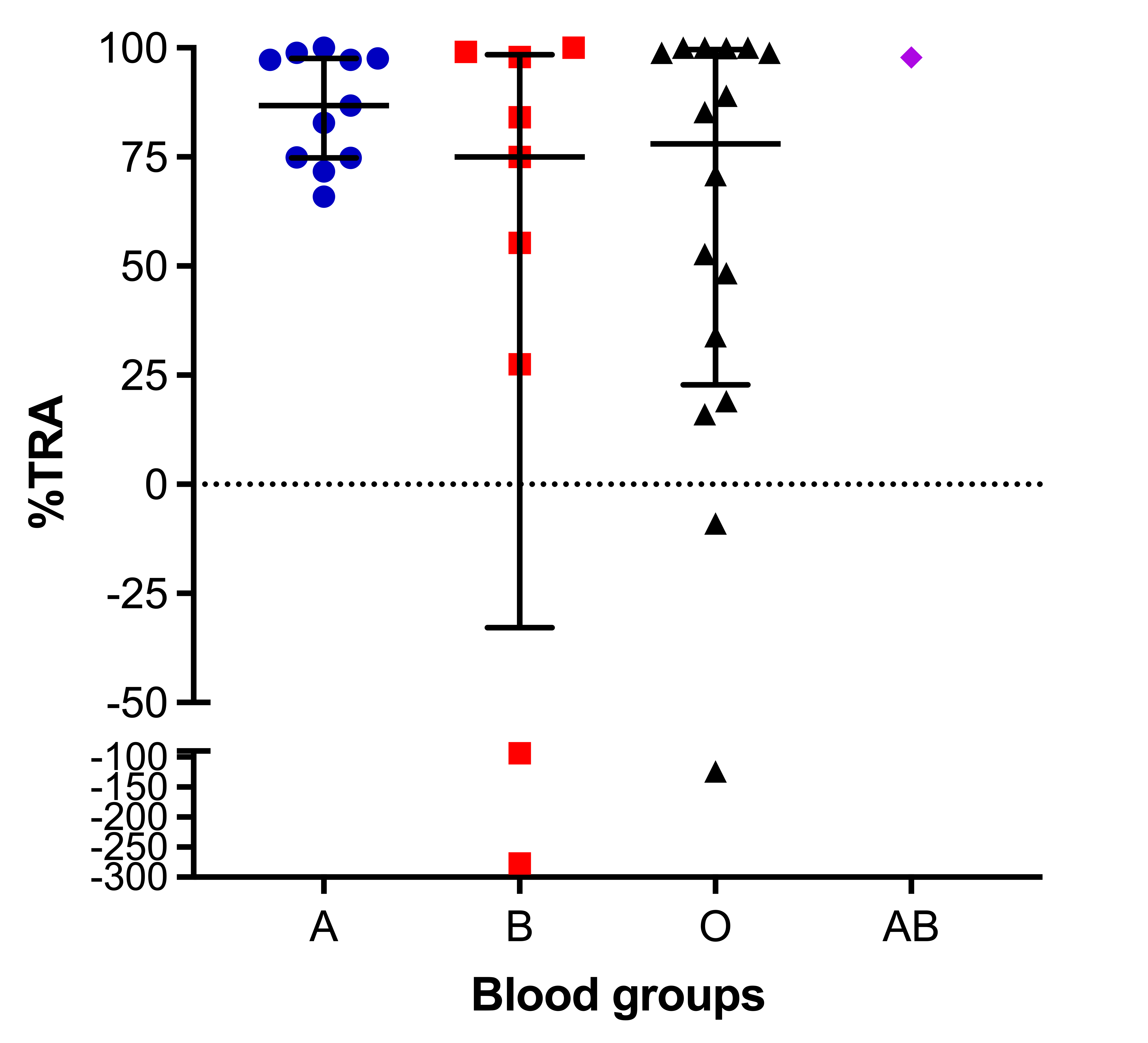

Supplement: jiad469_Supplementary_Data [file jiad469_supplementary_data.zip › Supplementary_Figure_S3.png]

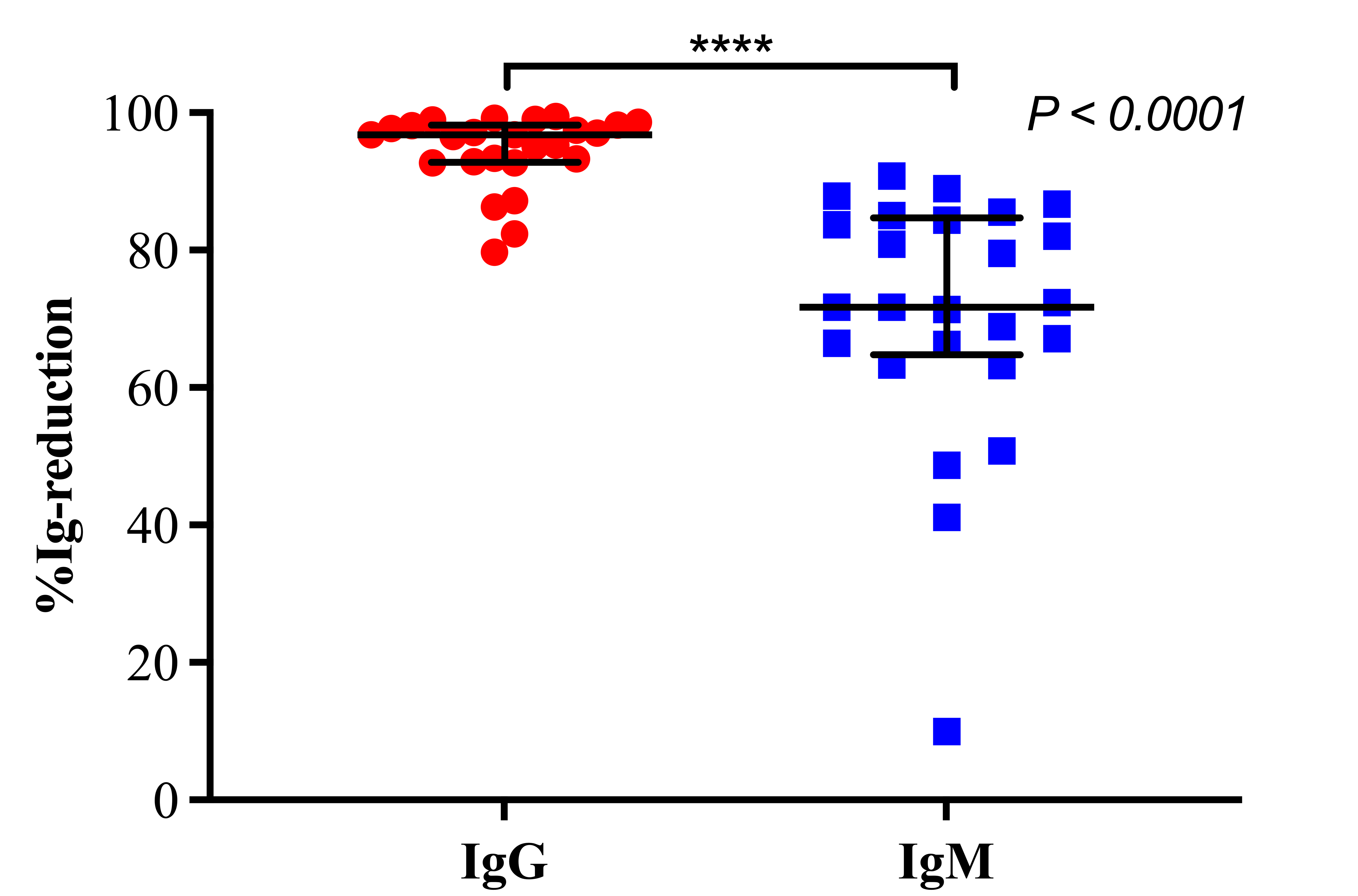

Supplement: jiad469_Supplementary_Data [file jiad469_supplementary_data.zip › Supplementary_Figure_S4.png]

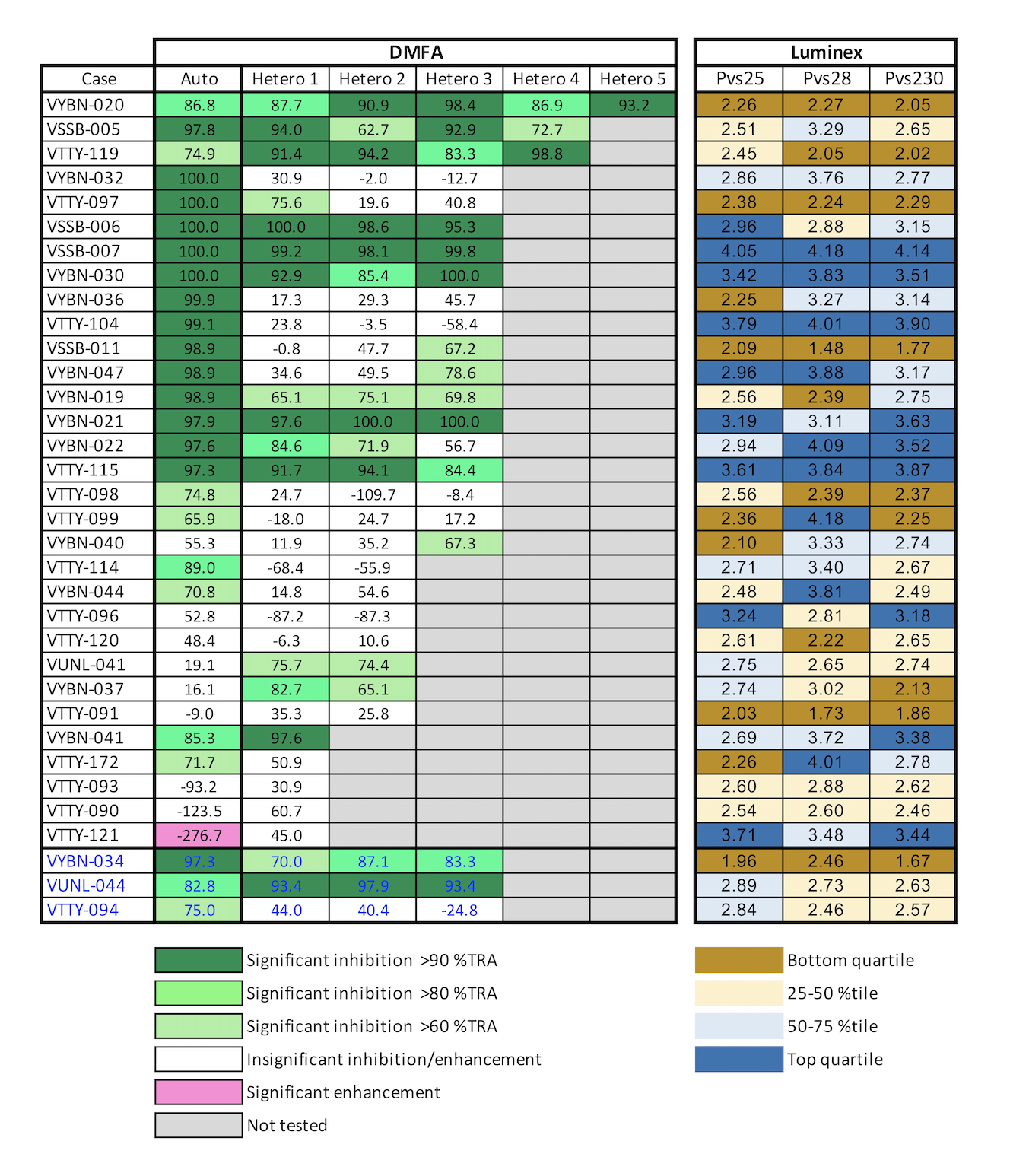

Supplement: jiad469_Supplementary_Data [file jiad469_supplementary_data.zip › Supplementary_Figure_S5.png]
